# Therapeutic Truths

**Published:** 1903-03

**Authors:** 


### Therapeutic Truths.

Mountain air and sea breezes are nature's specifics for hay fever.

Zinc sulphocarbolate, grain one-half, will check vomiting with surprising rapidity.

A large cupful of hot water drank every half-hour persistently, has cured severe cases of delirium tremens.

In the treatment of alcoholic habits, don't forget the necessity for excessive nutrition of some kind easily appropriated.

The compound tincture of benzoin is an admirable remedy for chapped hands, lips, cracked nipples, and the like.

Strychnia is an excellent remedy for uterine hemorrhage from atonicity or inertia. It may be given in advance if such a condition is anticipated.

There is nothing that so promptly cuts short congestion of the lungs, sore throat or rheumatism, as hot water when applied promptly and thoroughly.

To remove foreign bodies in the ear, dip the end of a camel's hair brush in glue and leave it in position against the body. When dry, after a few hours, pulling upon the brush will remove the whole thing.—*Canadian Practitioner.*

